# Quantifying Color Variegation in Melanoma Lesions Using Shannon Entropy

**DOI:** 10.7759/cureus.94779

**Published:** 2025-10-17

**Authors:** Jesús Iván Martínez-Ortega, Fatima Guadalupe Ramirez Ibarra, Ilse Fernández-Reyna, Alejandra Nicole Macias Quiroga

**Affiliations:** 1 Histology, Autonomous University of Nuevo León, Monterrey, MEX; 2 Dermatology, Dermatology Institute of Jalisco, Zapopan, MEX; 3 Faculty of Medicine, Autonomous University of Querétaro, Querétaro, MEX; 4 Mycology, Dermatological Center of Yucatan, Mérida, MEX; 5 Dermatology, Hospital Obrero No. 1, La Paz, BOL

**Keywords:** color variegation, dermoscopy, digital health, image analysis, melanoma, quantitative imaging, shannon entropy, skin cancer detection, smartphone photography, teledermatology

## Abstract

Color variegation is a key dermoscopic sign for melanoma, but lacks objective measurement. We computed Shannon entropy (H) from histograms of smartphone images (iPhone 13 Pro Max) of a histopathologically confirmed superficial spreading melanoma, a benign melanocytic nevus, and their perilesional skin as controls. Relative entropy (ΔH) was calculated to account for baseline skin heterogeneity. Absolute H values were 6.94 (melanoma), 6.67 (nevus), 5.75 (elderly perilesional skin), and 4.39 (young perilesional skin), with ΔH of +1.19 and +2.28, respectively. Entropy differentiated lesions from healthy skin, while ΔH highlighted baseline variability. Limitations include the single-case design, manual region-of-interest delineation, and non-standardized smartphone imaging without controlled lighting or dermoscopic magnification. Shannon entropy provides a reproducible, quantitative measure of color variegation, with potential applications in teledermatology and low-resource settings. Future studies should assess reproducibility across larger cohorts and standardized imaging modalities.

## Introduction

Cutaneous melanoma remains a major public health concern worldwide. In 2022, an estimated ~330,000 new cases were diagnosed, and 1.26 million people were living with a melanoma diagnosis in the previous five years, with marked regional variation by skin phototype distribution. Incidence is projected to reach ~510,000 new cases by 2040, underscoring the need for scalable, early-detection strategies [1,2]. Color information is central to both clinical and dermoscopic assessment. Features such as blue-white veil, shiny white structures, and regression patterns (gray dots and white areas) are recognized diagnostic cues [1]. Among these, polychromia or color variegation within a melanocytic lesion is one of the most reproducible and sensitive dermoscopic signs. Previous observational and meta-analytic studies have confirmed its importance, reporting sensitivities around 85-90% and specificities near 70-80% for melanoma detection [3,4]. Yet, interpretation remains largely visual and subject to interobserver variability, despite international consensus efforts to standardize terminology [5]. Chromatic patterns vary across melanoma subtypes and skin phototypes; acral, mucosal, and darker-skin lesions may differ from superficial spreading melanomas in lighter skin. Quantitative methods adaptable to such diversity could improve reproducibility across populations.

Computational approaches have attempted to formalize chromatic assessment. Early pipelines explored relative-color descriptors from clinical photographs [6], while dermoscopic research introduced entropy-based texture analysis to capture heterogeneity [7]. However, these methods often rely on specialized segmentation or model training, limiting clinical translation. Smartphone images were used in this study to test accessibility in low-resource settings, with dermoscopic validation planned for future studies. This technical report presents a proof-of-concept workflow that quantifies color variegation as Shannon entropy (H) computed from the lesion’s region of interest (ROI) histogram in ImageJ. By comparing a superficial melanoma, a benign melanocytic nevus, and their respective perilesional healthy skin, we aim to demonstrate the feasibility, reproducibility, and the added value of internal controls (ΔH) in adjusting for baseline cutaneous heterogeneity. The goal is not to define a threshold but to illustrate a low-cost, open, and replicable method that transforms subjective impressions of color into a continuous metric suitable for future validation and integration with teledermatology.

## Technical report

Methodology

Study Design

**Figure 1:**
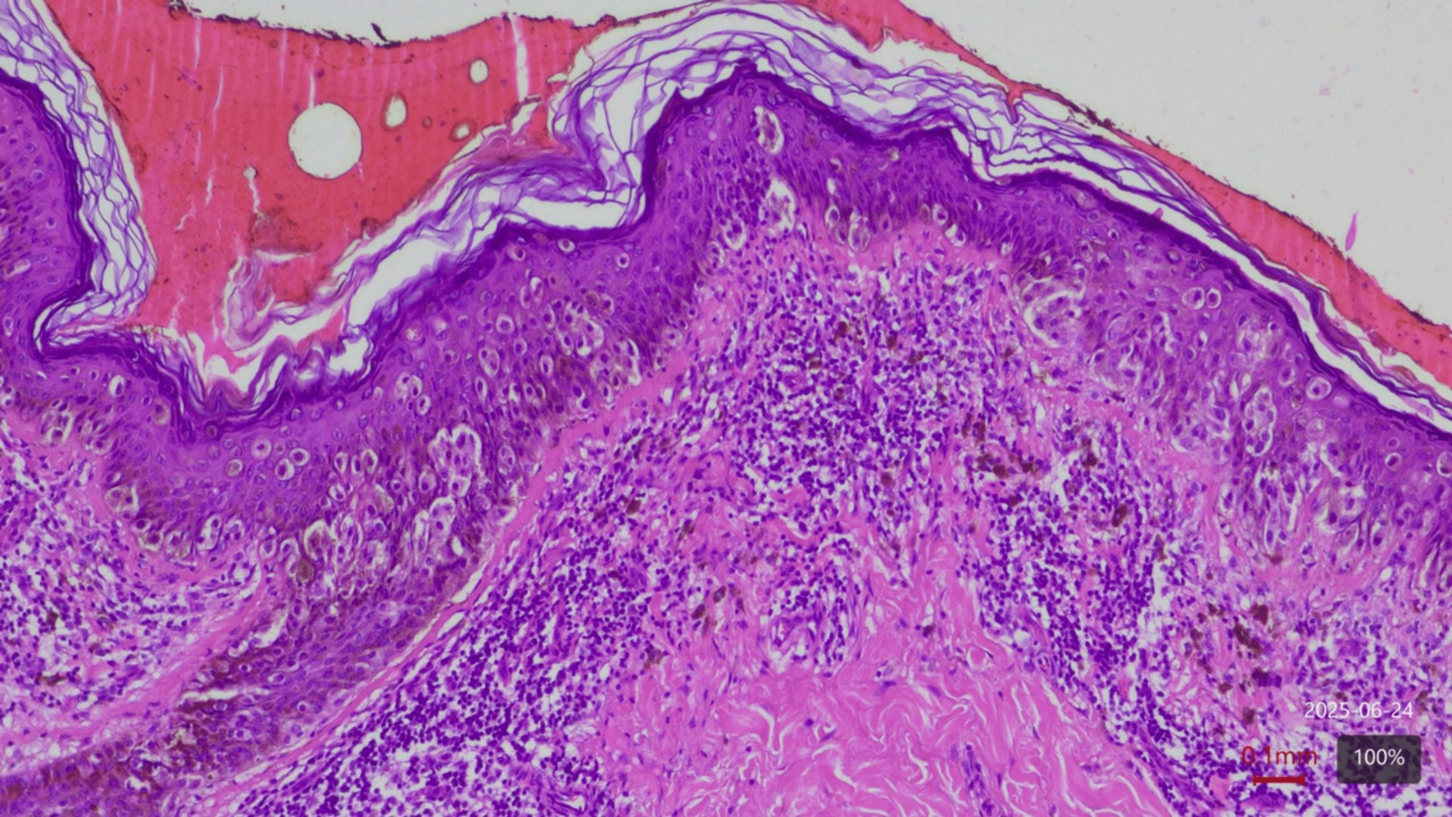
Histopathology of invasive superficial spreading melanoma. Hematoxylin and eosin, 10× magnification. The image shows a superficial spreading melanoma in the vertical growth phase. Atypical melanocytes form nests along the dermoepidermal junction and extend into the papillary dermis. Ulceration is absent. Breslow thickness is 0.5 mm, Clark level II. No lymphovascular or perineural invasion is observed. Mitoses are not evident. Tumor-infiltrating lymphocytes are moderately present. No signs of regression or microsatellites are noted. Surgical margins are clear, and the lesion was completely excised without recurrence. This histological specimen corresponds to the clinical lesion analyzed for chromatic entropy in the present technical report.

We present a proof-of-concept technical report to evaluate the feasibility of quantifying chromatic heterogeneity of melanocytic lesions using Shannon entropy (H) applied to image histograms. Three ROIs were analyzed, namely, a superficial melanoma and a benign melanocytic nevus, both confirmed by histopathology (Figure 1), alongside perilesional healthy skin as an internal control for each case. This study used anonymized images, and informed consent was obtained from patients. It was exempt from institutional review board approval due to its retrospective, non-interventional nature.

Image Acquisition

Images were acquired using an iPhone 13 Pro Max (12-megapixel camera, automatic white balance, no filters) under ambient homogeneous lighting. This non-professional setup was intentionally chosen to test the feasibility of a low-cost and reproducible workflow suitable for teledermatology and low-resource settings.

ROI Selection and Histogram Generation

Each ROI (lesion and adjacent healthy skin) was manually delineated using Fiji (ImageJ 1.53t, NIH, Bethesda, MD, 2023) with the polygonal selection tool. Grayscale histograms (0-255 intensity levels) were generated using the native Histogram plugin. Pixel counts were exported for further analysis.

Chromatic Entropy Calculation

Pixel frequencies were converted to normalized probabilities (p_i_). Shannon entropy was computed in bits as: \begin{equation} H = - \sum_{i} p_{i} \log_{2}(p_{i}) \label{eq:entropy} \end{equation}

where p_i_ is the relative probability of each gray-level intensity within the ROI.

Internal Controls and Relative Entropy

To minimize interindividual variability, relative entropy (ΔH) was defined as the difference between lesion entropy and perilesional skin entropy from the same patient. This approach adjusts for baseline cutaneous heterogeneity related to age, phototype, and actinic damage.

Objective

No diagnostic thresholds or inferential analyses were proposed. The aim was to demonstrate the feasibility and reproducibility of entropy (H) and relative entropy (ΔH) as objective metrics for the “C” (Color) component of the ABCD dermoscopy rule.

Results

**Figure 2:**
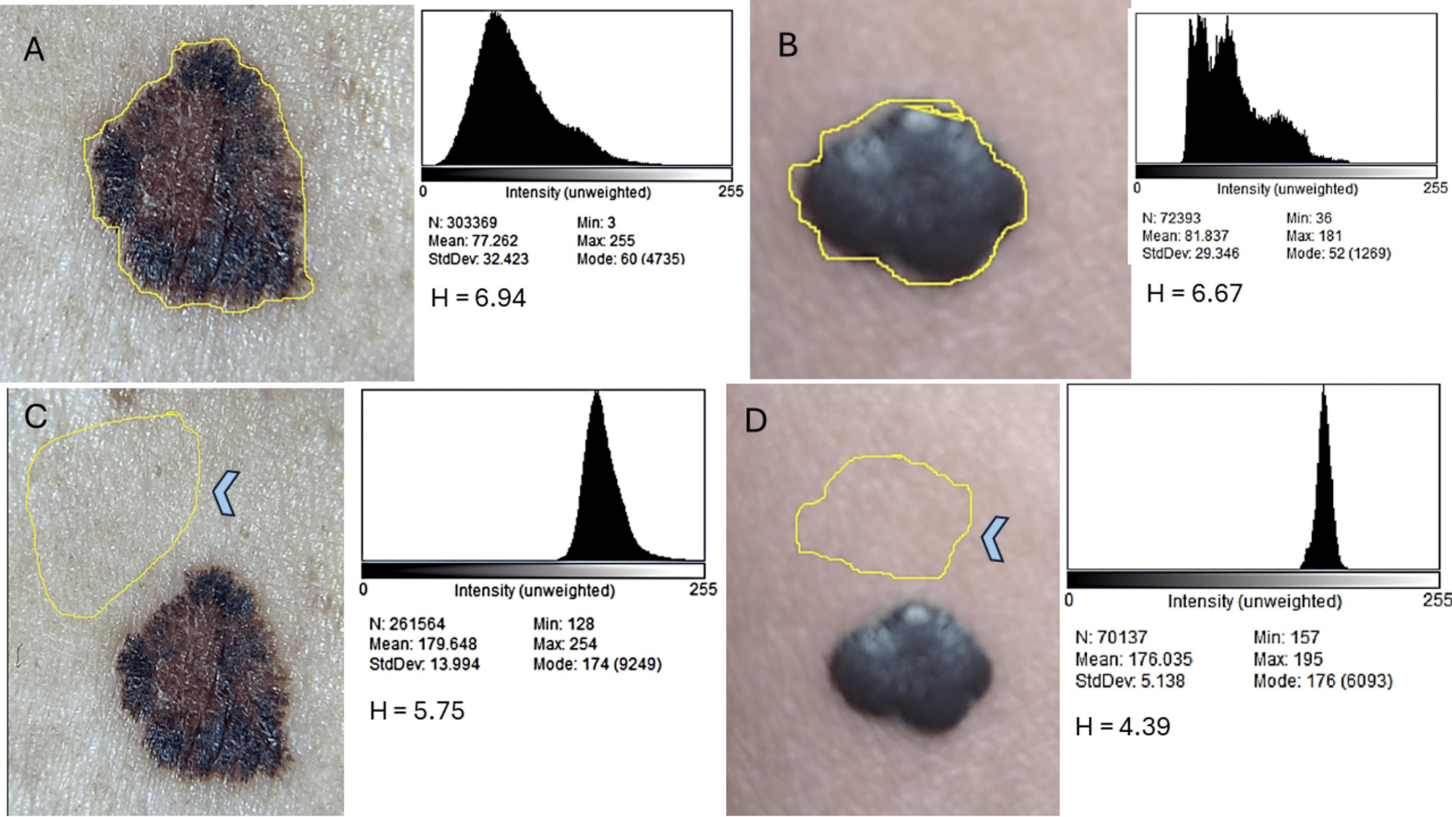
Regions of interest and corresponding histograms. (A) Superficial melanoma (H = 6.94 bits). (B) Benign melanocytic nevus (H = 6.67 bits). (C) Perilesional skin from an elderly patient (H = 5.75 bits). (D) Perilesional skin from young patient (H = 4.39 bits). Higher entropy (H) values indicate greater color heterogeneity within the lesion, distinguishing melanoma from benign and perilesional controls.

A superficial melanoma, a benign pigmented nevus, and their respective perilesional healthy skin were analyzed. Entropy values were computed for each ROI according to the Shannon formula:

\[ H = - \sum_{i=1}^{n} p_i \log_{2}(p_i) \]

where p_i_ is the normalized probability of each intensity level (Figure 2, Table 1).

**Table 1:** Entropy values for melanocytic lesions (superficial melanoma and benign nevus) and the corresponding perilesional skin. Melanoma showed the highest H, consistent with increased chromatic heterogeneity compared with benign and normal skin.

ROI Type	ROI Pixels	Absolute Entropy H (bits)	Relative Entropy ΔH (vs. Perilesional Skin)
Superficial Melanoma	303,369	6.94	+1.19
Perilesional Skin (Elderly Patient)	261,564	5.75	—
Benign Melanocytic Nevus	72,393	6.67	+2.28
Perilesional Skin (Young Patient)	70,137	4.39	—

The superficial melanoma demonstrated the highest absolute entropy (6.94 bits), consistent with marked color variegation. The benign nevus also exhibited high entropy (6.67 bits). However, when adjusted relative to perilesional healthy skin, the nevus showed a greater ΔH (+2.28 bits) than the melanoma (+1.19 bits). This difference was driven by the higher baseline heterogeneity of the elderly patient’s skin (5.75 bits) compared to the younger patient’s skin (4.39 bits).

Overall, these findings suggest that Shannon entropy can differentiate homogeneous healthy skin from pigmented lesions. In absolute terms, melanoma exhibits greater chromatic heterogeneity than the nevus. Additionally, relative entropy (ΔH) emphasizes the influence of baseline skin heterogeneity, highlighting the importance of using internal controls when interpreting chromatic metrics.

## Discussion

Dermoscopy increases diagnostic accuracy, reduces unnecessary biopsies, and improves the benign-to-malignant ratio in melanoma workups. A recent meta-analysis confirmed a pragmatic hierarchy of dermoscopic findings: pseudopods and shiny white structures serve as highly specific “rule-in” features, while irregular pigmentation and blue-white veil contribute more to sensitivity. Within this context, polychromia remains a high-yield sign, sensitive but not specific, making its objective quantification an attractive research target [3].

In our proof-of-concept, Shannon entropy successfully differentiated homogeneous skin from pigmented lesions, with values rising from 4.39-5.75 bits (perilesional skin) to 6.67-6.94 bits (nevus and melanoma). Importantly, melanoma displayed the highest absolute entropy, consistent with marked color variegation. However, when entropy was adjusted relative to each patient’s perilesional skin (ΔH), the benign nevus showed a larger increment than melanoma (+2.28 vs. +1.19 bits). This paradox is explained by baseline heterogeneity: the elderly patient’s perilesional skin exhibited higher entropy (5.75 bits) due to xerosis and photodamage, while the younger patient’s skin was more homogeneous (4.39 bits).

These results emphasize two insights: (1) entropy provides a sensitive and objective measure of chromatic heterogeneity, with melanoma showing the highest absolute entropy (6.94 bits) despite being an early-stage lesion imaged under non-standardized conditions; and (2) internal controls (ΔH) remain essential, as baseline skin heterogeneity substantially influences entropy values. Far from being a limitation, this proof-of-concept suggests that entropy is robust enough to detect variegation even in small, early melanomas captured with a smartphone camera. It is reasonable to expect that more advanced melanomas, which often display regression, blue-white veil, and additional chromatic complexity, would yield even higher entropy values, further supporting the metric’s potential for clinical translation.

Several computational approaches reinforce our findings. Entropy-based descriptors have reported high area under the curve in differentiating melanoma from nevi [7], and a convolutional neural network trained on color histograms can achieve comparable performance to traditional dermoscopy [8]. Our minimal workflow complements these efforts by providing a transparent, reproducible, and low-cost metric that could be integrated into more complex models.

The current study has several limitations. The single-case design, restricted to one superficial melanoma and one benign melanocytic nevus, limits the generalizability of findings. Manual delineation of ROIs introduces subjectivity that may influence entropy calculations. Image acquisition relied on non-standardized smartphone photography, without controlled illumination, color calibration, or dermoscopic magnification, which may reduce precision compared to dermoscopy or other standardized imaging modalities. The absence of dermoscopic images, while reflecting the focus on accessibility, limits comparability with established diagnostic standards. Finally, this proof-of-concept was confined to a superficial spreading melanoma in lighter skin; acral, mucosal, and darker-skin melanomas were not assessed and will require validation due to their distinct pigmentation patterns.

## Conclusions

Quantifying color variegation with Shannon entropy using open-source tools is feasible and potentially clinically relevant. This pilot proof-of-concept demonstrates that the method can provide a reproducible index of chromatic heterogeneity, complementing established dermoscopic features. Future application to dermoscopic datasets could enhance precision through standardized lighting and magnification, while smartphone-based acquisition supports teledermatology in low-resource settings. Integrating entropy metrics into artificial intelligence-assisted mobile workflows may ultimately facilitate remote evaluation and early melanoma detection, pending validation in larger and more diverse cohorts. Absolute (H) and relative (ΔH) entropy capture lesion complexity and baseline skin heterogeneity, illustrating the potential of accessible, quantitative approaches for future diagnostic development.
